# Modifying the Morphology of Silicon Surfaces by Laser Induced Liquid Assisted Colloidal Lithography

**DOI:** 10.3390/ma10111306

**Published:** 2017-11-14

**Authors:** Magdalena Ulmeanu, Robert L. Harniman, Petko Petkov, Michael N. R. Ashfold

**Affiliations:** 1 School of Chemistry, University of Bristol, Bristol BS8 1TS, UK; rob.harniman@bristol.ac.uk (R.L.H.); mike.ashfold@bristol.ac.uk (M.N.R.A.); 2 Cardiff School of Engineering, Cardiff University, Cardiff CF24 3AA, UK; PetkovPV@cardiff.ac.uk; 3 School of Engineering, University of Portsmouth, Portsmouth PO1 3DJ, UK

**Keywords:** colloids, laser processing, patterning, liquid induced photochemistry

## Abstract

Single, or isolated small arrays of, spherical silica colloidal particles (with refractive index *n*_colloid_ = 1.47 and radius *R* = 350 nm or 1.5 μm) were placed on a silicon substrate and immersed in carbon tetrachloride (*n*_liquid_ = 1.48) or toluene (*n*_liquid_ = 1.52). Areas of the sample were then exposed to a single laser pulse (8 ps duration, wavelength λ = 355 nm), and the spatial intensity modulation of the near field in the vicinity of the particles revealed via the resulting patterning of the substrate surface. In this regime, *n*_colloid_ < *n*_liquid_ and the near-field optical intensification is concentrated at and beyond the edge of the particle. Detailed experimental characterization of the irradiated Si surface using atomic force microscopy reveals contrasting topographies. The same optical behavior is observed with both liquids, i.e., the incident laser light diverges on interaction with the colloidal particle, but the resulting interaction with the substrate is liquid dependent. Topographic analysis indicates localized ablation and patterning of the Si substrate when using toluene, whereas the patterning induced under carbon tetrachloride is on a larger scale and extends well below the original substrate surface—hinting at a laser induced photochemical contribution to the surface patterning.

## 1. Introduction

The engineering of well-defined micro- and nano-scaled topographies on the surface of different materials, e.g., semiconductors, biomedical metals and polymers, has been explored as a strategy to control optical, tribological, wettability and biological properties. Many different top-down and bottom-up nanofabrication technologies (e.g., self-assembly processes, chemical etching, nano-imprinting, photolithography, electron beam and nanosphere lithographies) have been demonstrated in the quest to engineer such surfaces [1,2,3].

Colloidal lithography offers an attractive alternative to expensive, time consuming, low-throughput fabrication methods that are generally restricted to producing regular patterns. Colloidal lithography can be employed to produce a wide variety of in-plane nanoscale topographies, with irregular or lightly defined patterns, across large areas. Originally developed as a method of replicating sub-macroscopic patterns [4] and pioneered as a ‘natural lithography’ technique [5,6], colloidal lithography has since expanded into the electronics field as a means of producing single electron device structures [7], and has attracted interest in a biological capacity as a means of fabricating model nanostructured biomaterial surfaces [8]. For these applications, the nanotopographic profile should extend across a large surface area (ensuring repeatability of experiments and patterning of surfaces), be reproducible (allowing consistency in experiments) and, preferably, readily accessible (limiting the need for specialist equipment). Colloidal lithography techniques fit these criteria, when using size-selected nanoparticles in combination with a substrate to produce in-plane nanotopographies [9]. Colloidal lithography relies on the use of colloidal crystals (i.e., close packed arrays of spherical colloidal particles) as masks for etching and deposition. The feature size can be readily tuned by varying the diameter of the particles used in the mask. The feature shape can be tailored by varying the crystal structure of the mask, by etching the mask and by stepwise manipulation of the mask registry.

An ingenious use of the colloidal mask arises from the fact that a regular two-dimensional (2D) array of small particles can act as a lens array capable of converting an incident laser beam into a multiplicity of enhanced optical ‘hot-spots’, in parallel, that come to a focus in the near-field. The efficiency is extremely high. Having prepared the array, millions (billions) of nanostructures can be fabricated by irradiating with just a single laser pulse, making it ideal for large-area surface nanofabrication [10]. The enhanced field in this configuration is confined to localised areas defined by the particle size and packing, and is thus capable of yielding nanosized modifications on a substrate. The optical field enhancement mechanism in the case of transparent dielectric particles can involve contributions from the lens effect and/or from Mie scattering, depending on the particle size [11]. Using such methods, nanosize arrays of holes have been fabricated in a silicon surface by laser irradiation in air, mediated by hexagonally arrayed polystyrene particles with different diameters [12]. The colloidal mask can also be immersed in a liquid solution during processing, offering the possibility of controlling chemical or biological reactions on the nanoscale. The effective focal length of such a lens array is much extended (cf. in air) when immersed in a liquid like water [13]. Thus, the multiple foci that arise when a suitable microsphere array is illuminated by a femtosecond (fs) laser can be tuned to submerged locations (i.e., to positions beneath the substrate surface). The fabrication of different micro/nano-structures (e.g., nanometer-high annular bumps, convex bumps, etc.) on a glass surface has also been demonstrated, over large areas and without cracks and debris, by judicious variation of the incident laser fluence [13].

Our recently demonstrated Laser Induced Liquid Assisted Colloidal (LILAC) lithography technique [14,15] allows complex patterning of substrates using single laser pulses and isolated, or small arrays of, colloidal particles immersed in a liquid. Two distinct contributions to the patterning were identified: (a) near-field laser ablation (NF-LA) rings, caused by near-field intensity enhancement effects, and (b) near field scattering (NF-S) ring structures with characteristic minima and maxima that arise as a result of interference between the incident laser beam, light reflected at the substrate surface, and light scattered by the particle. The detailed surface topography was shown to depend on the difference in refractive indices of the particle (*n*_colloid_) and the liquid (*n*_liquid_), i.e., upon whether the incident light converges (*n*_colloid_ > *n*_liquid_) or diverges (*n*_colloid_ < *n*_liquid_) upon interaction with the particle. Here we report an in-depth study of the surface patterning of a silicon substrate using silica particles and either toluene or carbon tetrachloride as the liquid. *n*_colloid_ < *n*_liquid_ in both cases, and NF optical intensification is concentrated at and beyond the periphery of the particle. Nonetheless, despite their very similar optical behaviours, detailed characterization of the irradiated Si surface using atomic force microscopy (AFM) reveals clear liquid-dependent differences in the scale and extent of the surface patterning. This study represents another step in progressing our understanding of the potential of the LILAC lithography method for nano-patterning and texturing Si surfaces, and for providing fundamental insights into short laser pulse–liquid interactions in the NF.

## 2. Results and Discussion

Here, we explore how the topographies induced by NF-LA and NF-S effects vary with the choice of liquid, with all other variables maintained as near constant as possible. Toluene (C_6_H_5_CH_3_, *n*_liquid_ = 1.52) and carbon tetrachloride (CCl_4_, *n*_liquid_ = 1.48) both have refractive indices larger than that of the silica particles (*n*_colloid_ = 1.47) used in the present work, thereby satisfying the condition *n*_colloid_ < *n*_liquid_ and confining the topographic patterning of the silicon substrates to areas outside the periphery of the particle [14].

**Figure 1:**
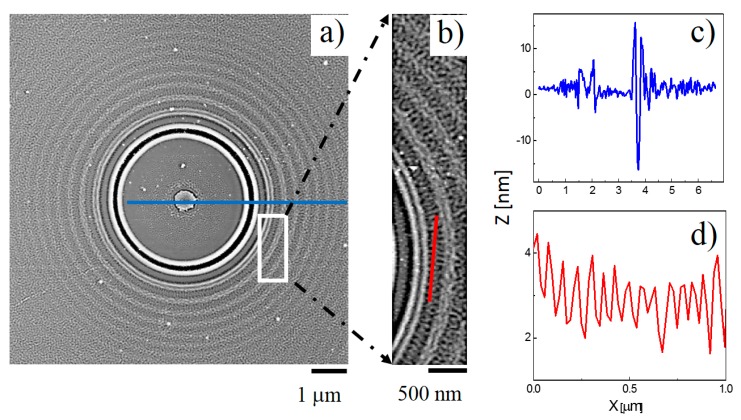
Plan view AFM images (overview (**a**) and expanded detail (**b**)) of the near-field pattern imprinted on a Si substrate by single shot LILAC lithographic processing using a single colloidal particle with *R* = 1.5 μm and *n*_colloid_ = 1.47 in C_6_H_5_CH_3_ (*n*_liquid_ = 1.52) and an incident fluence *F* = 5.6 J∙cm^−2^. The radial and angular periodicities within the so-formed rings are revealed via the cross-sectional profiles along the blue line in (**a**) and the red line in (**b**) that are shown in, respectively, (**c**) and (**d**).

The patterns shown in Figure 1 were produced when a single 355 nm laser pulse (with peak fluence, *F*_peak_ = 5.6 J∙cm^−2^) was incident from above on an isolated silica particle, with radius *R* = 1.5 μm, sitting on a Si substrate immersed in C_6_H_5_CH_3_. The plan view AFM images (Figure 1a,b) and the cross-sectional profiles (Figure 1c,d) reveal an obvious NF-LA ring surrounded by further annular NF-S rings characterised by a wrinkled topography. The colloidal particle is removed by the one-shot exposure, but its location immediately prior to irradiation is marked by a small bump (height ~ 5 nm) on the substrate surface at a position directly under the particle centre. The observation of this localised feature implies that the substrate under the particle is not totally immune to the irradiation process. NF-LA effects are responsible for the innermost, dark ring and cross-sectional AFM analysis shows it to be bounded by elevated ridges. The radial separation between the centres of the NF-LA ring and the first NF-S ring is ~450 nm. The periodicity of these surface patterns, ~250 nm, is tighter than that observed when ablating in air, reflecting the reduced effective wavelength of the laser light (λ = λ_0_/*n*_liquid_). The ring pattern is also less extensive than that formed when ablating in air, reflecting the faster decline in the scattered field intensity with increasing *R* at higher *n* [15].

As the expanded view in the Figure 1b shows, the outer NF-S rings and the intervening annular regions exhibit complex surface ripple structures, with a discernible periodicity in the angular coordinate within each ring. The cross-sectional AFM analysis shows a periodicity Λ ~ 70 nm, with half-pitch resolution as small as 30 nm, indicating that these belong to the class of deep-sub-wavelength ripples (DSRs) with a period to laser wavelength ratio in the domain Λ/λ < 0.4 [16]. The average ripple height of these DSRs, an important parameter in the field of nano-etching, is ~2 nm (formed in a single-shot exposure).

**Figure 2:**
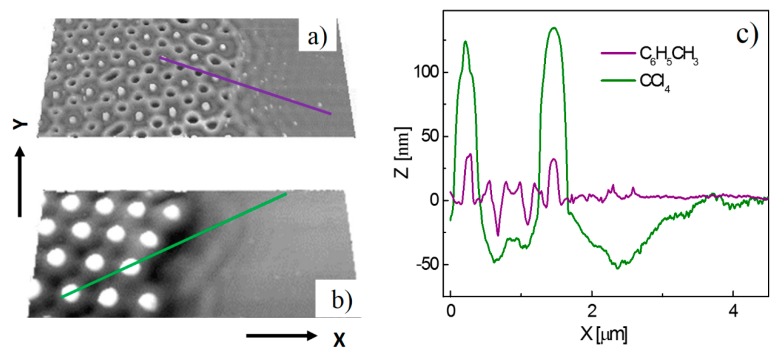
Plan view AFM images (overview (**a**,**b**)) illustrating selected patterns formed by 355 nm single-pulse illumination (*F*_max_ = 5.6 J∙cm^−2^) of a Si substrate surface supporting isolated clusters of colloidal particles with *R* = 350 nm and *n*_colloid_ = 1.47 in (**a**) C_6_H_5_CH_3_ and (**b**) CCl_4_. The *X* and *Y* axes correspond to the lengths X = 6.5 μm and Y = 2.9 μm, respectively. The topographical changes induced in the silicon substrate are revealed by the cross-sectional profiles shown in (**c**).

The interference of the NF-LA and NF-S rings formed during LILAC lithographic processing with a close packed array of colloidal particles (rather than just an isolated particle) can induce a regular 3-D nanotexture. This is illustrated in Figure 2a,b, which show AFM images of Si surfaces, both of which hitherto supported localised close-packed monolayer arrays of silica particles with *R* = 350 nm, and were formed by exposure to a single 355 nm laser pulse with peak fluence, *F*_peak_ = 5.6 J∙cm^−2^. The key distinguisher is the choice of solvent used in the LILAC processing: C_6_H_5_CH_3_ for the surface patterning in Figure 2a, CCl_4_ in the case of Figure 2b.

The substrate was continually translated during these experiments, ensuring that the irradiated areas were equally spaced, but there is no necessity that these processed areas be centered on regions covered by localized colloidal arrays. Areas chosen for particularly close analysis in the present work were those where the center of the (near-Gaussian) laser intensity profile straddled the boundary between masked and bare regions of the substrate. Figure 2a,b shows local maxima (white) corresponding to the centers of the particles in the close packed array that constituted the colloid mask prior to LILAC processing in C_6_H_5_CH_3_ and CCl_4_, respectively. As noted previously, the observation of these localized features implies that the substrate under the particles is affected by the irradiation process. Our previous high-resolution transmission electron microscopy analysis of these features indicated the polycrystalline nature of the Si in this region [14]. Cross-sectional AFM analysis (Figure 2c) shows these maxima peaking ~25 nm above the neighboring surface. The rest of the surface profiling is again concentrated just outside the area eclipsed by the colloidal particles (as expected, given *n*_colloid_ < *n*_liquid_) and can be understood as the superposition of, primarily, the NF-LA rings associated with neighboring particles. The largest craters (the black features in Figure 2a, attributable to the overlap of NF-LA rings from three adjoining particles) appear as a hexagonal lattice, reflecting the close packing in the original colloidal mask.

Cross-sectional AFM analysis also allows investigation of differences in line scan profiles from areas of the substrate processed with and without the presence of the colloidal mask. Height zero in these line scans is taken to be the Si substrate surface in regions remote from that which was covered by colloidal particles during laser irradiation, and thus show no patterning. As Figure 2c shows, the craters formed in the interstices between abutting particles by single laser-shot processing through the colloidal mask immersed in C_6_H_5_CH_3_ are ~25 nm deep and ~200 nm full width at half maximum (FWHM). The same hexagonal symmetry is clearly evident in the surface topography of the Si surface processed through the colloidal mask immersed in CCl_4_. However, as Figure 2c also shows, the height of the maxima defining the center locations of the particles in the mask and the depth and width of the craters formed in regions of overlap between the NF-LA and/or NF-S rings from adjacent particles are all much increased when processing under CCl_4_; the maxima project ~125 nm above the unprocessed surface, while the intervening craters have coalesced into single ~50 nm deep, double well features. The cross-sectional image also suggests localized ablation (removal) of the Si substrate to a depth of ~50 nm (at *F*_peak_ = 5.6 J∙cm^−2^) immediately outside the masked region. The patterning induced under CCl_4_ is on a larger scale, and extends well below the original substrate surface, hinting at some photochemical enhancement of the LILAC processing probability by (multi)photon excitation of the CCl_4_ solvent in regions of highest light intensity.

## 3. Materials and Methods

**Preparing the colloid mask**: All chemicals used in this study were obtained commercially and used as supplied. Aqueous suspensions of monodisperse (coefficient of variation of 10–15%) silica colloidal particles with mean radius, *R* = 350 nm or *R* = 1.5 μm (10% solid concentration, with a specified refractive index *n*_colloid_ = 1.47 were sourced from Bangs Laboratories.

The isolated small arrays of colloidal particles on the Si substrate were produced by spin coating. The step sequence used in the spin-coating process was as follows: (a) the substrates were cut from n-type Si wafers (Crystal GmbH, Weilheim, Germany) and pre-treated in an oxygen plasma (Femto Diener, 80 W power and 0.5 mbar O_2_ pressure) for 10 min to remove contaminants and render the surface hydrophilic; (b) 15 mL of the suspension containing the colloidal particles was dropped onto the Si substrate while spinning (2000 revolutions per minute) over a period of 60 s.

**Laser Processing**: Localized regions of the translating substrate were irradiated with a train of laser pulses from a diode-pumped Nd:YVO_4_ laser (Lumera Laser GmbH, 8 ps pulse duration, maximum power *P* = 2 W at λ = 355 nm). The beam was controlled by Wave Runner software (Nutfield Technology, Hudson, NH, USA) and a galvo-scanner combined with a telecentric lens with focusing distance *f* = 103 mm. Powers used in the present work was *P* = 100 mW, which translate into an incident fluence at the substrate surface of *F* = 5.6 J∙cm^−2^. The colloid coated Si substrate was completely immersed in the liquid of interest (volume ~ 1 cm^3^) in an open trough (of dimension 25 × 25 × 20 mm^3^) mounted on a motorised 2-(*X*, *Y*-) axis translation stage. Single-shot laser irradiation in the liquid of interest usually resulted in particle removal. Sample characterization involved use of optical microscopy and atomic force microscopy (Bruker Multimode AFM).

## 4. Conclusions

The surface patterning achieved with the LILAC lithographic technique when *n*_colloid_ < *n*_liquid_ originates from a combination of near and scattering fields (and their mutual interference) arising from individual colloidal particles. Patterns formed by single-pulse irradiation of Si substrates supporting both isolated particles and localized close-packed arrays of particles have been investigated for two different liquids, CCl_4_ and C_6_H_5_CH_3_. In the case of C_6_H_5_CH_3_, AFM analysis confirms that the overlap of NF-LA and NF-S fields when processing the Si surface through a close packed array of particles offers a route to creating grids of variable aspect ratio holes with sub-wavelength FWHM cross-sections. Equivalent studies using CCl_4_ as liquid yield analogous but topographically exaggerated features, hinting at a photochemical contribution to the surface patterning. The present study should serve to encourage further investigations of the potential opportunities and applications of the LILAC lithography technique.
